# Primitive Neuroectodermal Tumor of the Vulva in an Adolescent Girl

**DOI:** 10.14740/wjon819w

**Published:** 2014-12-03

**Authors:** Geetha Narayanan, Varun Rajan, Jaisankar Puthusseri, Jayasree Kattoor, Lali V. Soman

**Affiliations:** a Department of Medical Oncology, Regional Cancer Centre, Trivandrum 695011, India; b Department of Pathology, Regional Cancer Centre, Trivandrum 695011, India

**Keywords:** PNET, Vulva, Adolescent

## Abstract

Extraskeletal primitive neuroectodermal tumor (PNET) is rare and that affecting the female genital tract is extremely rare. We report the case of a 17-year-old girl who presented with swelling of the clitorus. She underwent excision and histopathology was PNET. She received chemotherapy and radiation and achieved complete remission. However, she relapsed locally and systemically soon, progressed and died.

## Introduction

Ewing’s sarcoma (ES)/primitive neuroectodermal tumor (PNET) is a high grade malignant neoplasm that mainly involves the skeleton. Primary extraskeletal PNET is rare, comprising 1% of soft tissue sarcomas [1]. PNET rarely affects the female genital tract and only a few cases of vulvar ES/PNET have so far been reported [2]. We report the case of an adolescent girl with PNET of the vulva.

## Case Report

**Figure 1:**
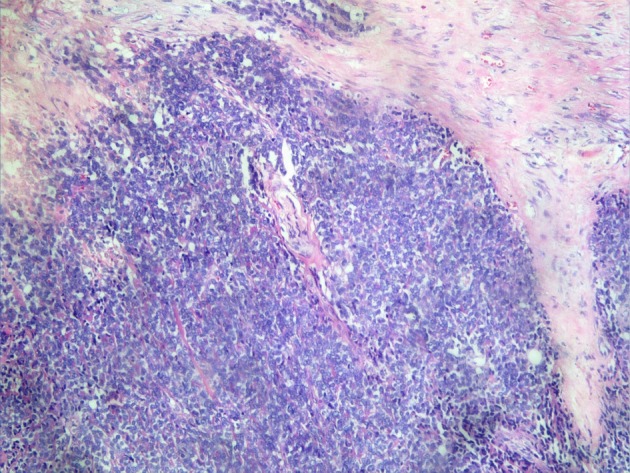
Round cells in large lobules separated by fibrocollagenous stroma (H&E ×20).

**Figure 2:**
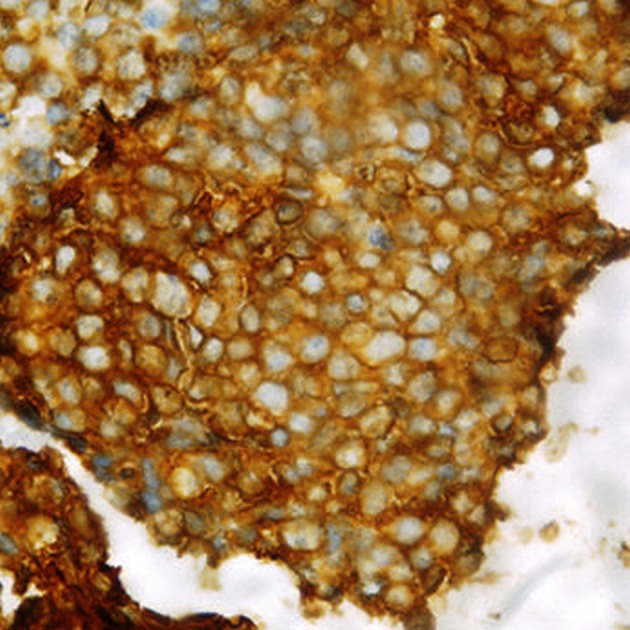
Section showing MIC2 positivity.

**Figure 3:**
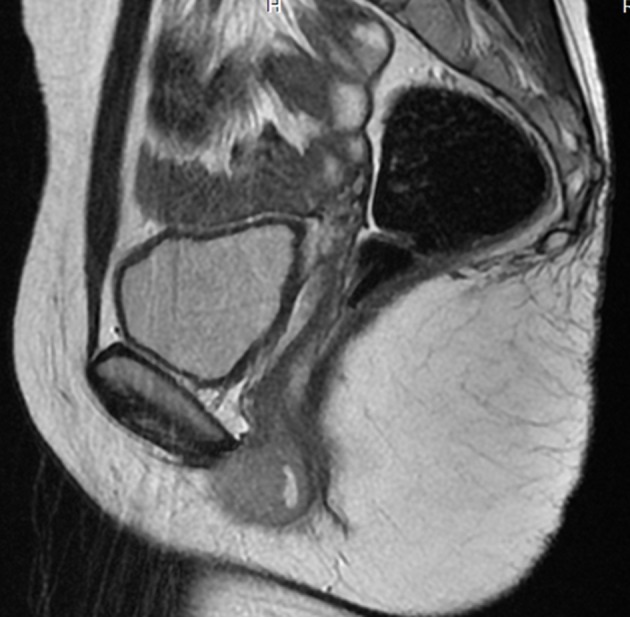
MRI T2WI SAG-lesion appears hyperintense.

**Figure 4:**
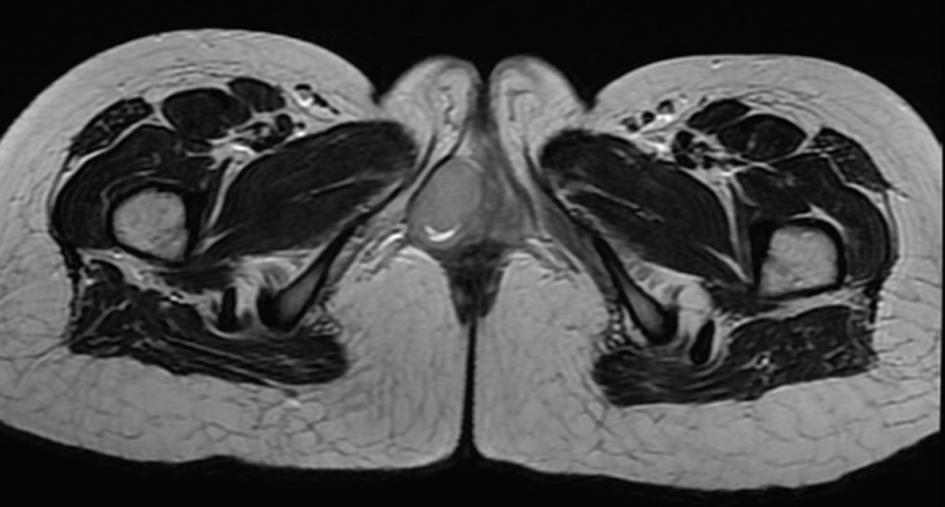
MRI axial T2WI-lesion appears hyperintense.

A 17-year-old girl presented with swelling at the clitorial region of few weeks duration. On examination, there was a swelling 3 × 2 × 2 cm at the clitorius. Computerized tomography (CT) scan of the abdomen and pelvis showed a right ovarian cyst of 3.5 × 2.5 cm in addition to the clitorial mass. She underwent diagnostic laparoscopy and left ovariectomy for the ovarian lesion and excision of the clitorial mass at the local hospital and was subsequently referred to us. Her performance status was 0, blood and serum chemistries were normal and lactate dehydrogenase was 405 U/L. The histopathological examination of the specimen from clitoris showed a neoplasm composed of round cells in large lobules separated by fibrocollagenous stroma, cells with scanty to moderate cytoplasm and vesicular nuclei, and many thin walled blood vessels were seen (Fig. 1). The tumor cells were strongly positive for MIC2, positive for neuron-specific enolase and negative for desmin, cytokeratin, myogenin and synaptophysin (Fig. 2). This was diagnostic of PNET/ES. The ovarian lesion was non-malignant. She was started on chemotherapy with vincristine, adriamycin, cyclophosphamide alternating with ifosphamide and etoposide and received local radiation 45 Gy/20# at ninth to 12th weeks. She achieved complete remission and chemotherapy was continued for 1 year. However, 4 months after treatment completion, she developed local recurrence in the form of well-defined mass 2 × 2 cm at the mons pubis. MRI showed multiple vulval deposits, deposits in the lower anterior pelvic wall, bilateral iliac bone, sacrum and femur (Fig. 3, 4). Bone scan showed multiple bone deposits and CT scan of chest showed multiple lung metastasis. She was given only supportive care and she died 6 months later.

## Discussion

PNET is considered an entity of Ewing’s family of tumors and has a similar natural history, prognosis, immunohistochemical and cytogenetic profiles [2]. They account for 4% of malignancies, of which 15% are of extraskeletal or soft tissue origin [3]. PNETs are classified into central and peripheral PNETs according to the cells of origin.

Extraosseous PNET is also now regarded as a member of the ES/PNET family. It typically involves the soft tissues of the chest wall, pelvis, paravertebral region, abdominal wall, retroperitoneal region and extremities of children, adolescents and young adults [4, 5]. PNETs of the female genital tract are rare, with the most common sites being the ovary and uterine corpus [2, 6]. However, involvement of cervix, vulva and vagina is exceedingly rare [7-10]. PNETs occur more commonly in the second decade of life. The age of previously reported cases of PNET of the vagina varied between 17 and 47 years old and our patient was 17 years.

PNET of the external genitalia usually presents as a rapidly growing mass, with watery and foul smelling discharge per vagina or with pressure symptoms such as tenesmus and difficulty in passing urine [2].

A 25-year-old girl with sickle cell disease who developed PNET of vagina, treated with surgery, radiotherapy and chemotherapy was alive at 1 year [7]. Cetiner et al reported two young adults with PNET arising in vulva treated aggressively and who survived for more than 7 and 4 years [11]. A 36-year-old lady with PNET of paravaginal region was treated with surgery, radiotherapy followed by adjuvant chemotherapy and was alive at 12 months [12]. McCluggage et al reported four cases of ES/PNET family involving the vulva or vagina which occurred in women aged 19, 20, 30 and 40 years and ranged in size from 3 to 8 cm. All cases were characterized by membranous CD99 staining and two exhibited nuclear FLI-1 positivity [10]. A case of paravaginal PNET occurring in a 29-year-old lady was also reported [13]. A 14-year-old girl with left vulvar mass which was excised followed by chemotherapy and radiotherapy died of pulmonary metastasis within 9 months of the initial surgery [14]. A 20-year-old female presented with a rapidly growing painful mass of the left labium majus and bone and lung metastasis [15]. Among the 11 cases of PNET of female genital tract reported by Xiao et al, two were of vulval origin [16].

PNETs are round cell tumors showing varying degree of neuro-ectodermal differentiation. Immunohistochemical studies help in differentiating PNET from other round cell tumors. Positivity for CD99, a product of MIC2 gene is useful in confirming the diagnosis but it is not specific. Other markers such as vimentin, FLI-1 protein, synaptophysin, neuron-specific enolase and S100 protein are also positive in PNET. Lack of desmin, myogenin and myoD1 can rule out rhabdomyosarcoma and LCA negativity excludes lymphoma [1, 9, 10]. In our patient also the tumor cells were strongly positive for MIC2, positive for neuron-specific enolase and negative for desmin, cytokeratin, myogenin and synaptophysin. ES and PNETs share a specific t (11; 22) (q24;q12) chromosomal translocation. The breakpoints involve the *EWS* gene on chromosome 22 and the *FLI-1* gene on chromosome 11. Vaginal PNETs were also found to have *EWS* and *FLI-1* genes by molecular study [9, 10].

Surgery, chemotherapy and radiotherapy are the treatment for PNET [2]. The chemotherapy used in the treatment of PNET of the vulva is similar to high-risk protocol for Ewing’s family of tumors and consists of combination chemotherapy with vincristine, cyclophosphamide, doxorubicin alternating with ifosfamide and etoposide. Surgery varies from tumor biopsy, wide local excision of the tumor and radical surgery [2, 17, 18]. Adjuvant radiotherapy is also used in the treatment of PNET of the vulva/vagina. Progress in the treatment of ES/PNET has improved the survival rate from 10% to 75% after the introduction of chemotherapy for patients with localized tumors [19]. The addition of ifosfamide and etoposide to a standard regimen of vincristine, doxorubicin, and cyclophosphamide significantly improved the outcome for patients with non-metastatic ES/PNET [20]. PNETs arising from extra osseous sites have an aggressive history and poor prognosis compared to osseous PNETs. Women with vaginal/vulval PNET were reported to be free from the disease at 18 - 48 months of follow-up [10, 17, 18].

Management of these patients involves multidisciplinary approach to improve the outcome and survival rate.
